# Physicochemical Properties and Mouthfeel in Commercial Plant-Based Yogurts

**DOI:** 10.3390/foods11070941

**Published:** 2022-03-24

**Authors:** Maija Greis, Taru Sainio, Kati Katina, Alissa A. Nolden, Amanda J. Kinchla, Laila Seppä, Riitta Partanen

**Affiliations:** 1 Department of Food and Nutrition, University of Helsinki, P.O. Box 66, 00014 Helsinki, Finland; taru.sainio@helsinki.fi (T.S.); kati.katina@helsinki.fi (K.K.); laila.seppa@helsinki.fi (L.S.); 2 Department of Food Science, University of Massachusetts Amherst, Amherst, MA 01003, USA; anolden@umass.edu (A.A.N.); kinchla@umass.edu (A.J.K.); 3 Valio Ltd., P.O. Box 10, FI-00039 Helsinki, Finland; riitta.partanen@valio.fi

**Keywords:** physicochemical properties, rheology, sensory evaluation, dynamic mouthfeel perception, plant-based yogurt alternative, oat

## Abstract

There is a growing need for plant-based yogurts that meet consumer demands in terms of texture. However, more research is required to understand the relationship between physicochemical and mouthfeel properties in plant-based yogurts. The purpose of this study was to determine the physicochemical properties of five commercial plant-based yogurt alternatives with different chemical compositions, making comparisons to dairy yogurts and thick, creamy, thin, and watery mouthfeel sensations. The physicochemical parameters studied included large and small deformation rheology, particle size, soluble solids, acidity, and chemical composition. Significant differences in flow behavior and small deformation rheology were found between dairy- and plant-based yogurts. Among plant-based yogurts thick, creamy, thin, and watery mouthfeel sensations were strongly associated with steady shear rates and apparent viscosity. The results highlight the importance of large deformation rheology to advance the use of plant-based ingredients in the development of yogurt alternatives. Furthermore, this study demonstrates that dairy- and plant-based yogurts with a similar mouthfeel profiles may have different viscoelastic properties, which indicates that instrumental and sensory methods should not be considered substitutive but complementary methods when developing plant-based yogurts in a cost-effective and timely manner.

## 1. Introduction

In terms of dairy alternatives, oat-based products are a popular substitute due to their mild flavor properties and potential positive health benefits [1]. The functional properties inherent to plant-based ingredients often include a lower gelling strength compared to animal-based systems; therefore, the gelling structures are enhanced through the use of hydrocolloids [2,3,4,5]. In previous work, we reported that the sensory properties among oat-based yogurts differ, some of them resembling their dairy counterparts, both in mouthfeel and pleasantness [6]. Due to the complexity and variety in the composition of these products, it is difficult to explain their mouthfeel differences through compositional factors alone. Therefore, rheology, with the help of acidity, soluble solids, and particle size measurements, was applied to better understand the mouthfeel sensations and pleasantness of these plant-based yogurts.

There is extensive prior literature exploring the relationship between the rheological properties and sensory attributes of dairy yogurts [4,7,8,9,10,11,12,13,14]. Other physicochemical parameters have also been successfully linked to mouthfeel in dairy yogurts. Particle size-related parameters have been shown to influence the creamy mouthfeel [14,15,16,17,18]. In addition, the reduction in sugar in dairy yogurt has been linked to a decrease in viscosity, resulting in a thin and watery mouthfeel [19]. According to another study, a watery mouthfeel is the opposite to a creamy one and relates to low-fat content in emulsion-filled gels [20].

An increasing number of studies are exploring the consumer acceptance and physicochemical properties of different plant-based yogurts [2,5,21,22]. A noteworthy study reports the rheological properties, sensory perception, and consumer acceptability of lactic acid fermented, oat-based gels [2]. They demonstrated that a gel with a higher total solids content was perceived as creamier compared to a gel with a lower total solids content. Another study reports the compositional and physicochemical properties with liking of different commercial plant-based yogurts [20]. They concluded that soy, coconut, and cashew yogurts scored similarly in terms of texture liking as dairy yogurts. A more recent study aims to understand the sensory acceptability and textural properties in Australian commercial dairy and plant-based samples [20]. The selected soy, coconut, and dairy yogurts showed wide variations in their microstructure and rheology. The results highlight that the protein content, gel firmness, and consistency coefficient displayed a positive relationship with overall liking [21]. Notably, these previous studies did not include oat-based yogurts in their experiments [21,22].

Our study aimed to determine the physicochemical properties of plant-based yogurts. The results were compared to dairy counterparts and previously studied mouthfeel properties. Our hypothesis is that oat-based structures are predominantly carbohydrate gels, and thus provide a more fine-stranded network compared to dairy yogurts. Instead, dairy yogurts provide a distinguished particle gels system attributed to the network of protein particles and protein-covered fat droplets. Our previous findings suggest that the dominant mouthfeel attributes perceived during the early stages of mastication have a larger impact on mouthfeel pleasantness than the dominant attributes perceived later during mastication [6]. Therefore, conventional rheological methods are expected to be relevant in determining factors that contribute to mouthfeel liking and disliking.

We will examine these questions using a variety of commercial products. They represent a wide range of mouthfeel properties that would not be achievable if using a simple, controlled model product. By choosing a set of unflavored commercial products from the same plant source, we limit the differences in flavor and thus focus only on the mouthfeel. In this experiment, our focus is on four following specific positive and negative mouthfeel sensations contributing to the liking of the products: thickness and creaminess (positive) and wateriness and thinness (negative) based on the findings in our previous study [6].

## 2. Materials and Methods

### 2.1. Samples

Five unflavored plant-based yogurt alternatives (P1-P5) and two unflavored dairy yogurts (D1-D2) were purchased from a local supermarket (Table 1) in Finland. The plant-based products were spoonable yogurt-like semisolid snacks labeled as “oat-based yogurts”. Dairy-based references included two spoonable dairy yogurts (fat contents of 2.5% and 4%). All samples were fermented with the help of an added starter. These yogurt alternatives were selected due to their different structures. In addition, they represent the variety of oat-based yogurt alternatives in the market. The reference samples resembled typical dairy yogurts in the market. The products were sourced in duplicate so that analysis could be split for sensory [6], and physicochemical analyses. All samples were stored at 5 °C prior to the sensory and physicochemical analyses. All samples were analyzed both in the sensory analysis and instrumental measurements at 10 °C within their declared shelf-life period. The studied yogurt alternatives are referred to as “plant-based” instead of “oat-based yogurts”, as they contain pea and potato protein in addition to oat protein. All instrumental measurements were performed in triplicate, apart from particle size assessment, where three separate measurements were conducted for each sample. A summary of the analysis is presented in Table 2.

### 2.2. pH and Titratable Acidity

The pH and titratable acidity (TTA) were analyzed from both sets of samples (sensory and instrumental) in order to confirm the statistical similarity between experiments. Total titratable acidity was analyzed using instrumental analysis: 10 g of each sample was homogenized (1 min) with 10 mL of acetone and 90 mL of Milli-Q water using a Bamix blender (Switzerland), as described in [23]. The TTA was determined as the amount of 0.1 M NaOH required to adjust the end pH of samples to 8.5. A pH meter (Model HI 99161, Hanna Instruments, Woonsocket, RI, USA) and TTA titrator (EasyPlus Titration, Mettler Toledo, Columbus, OH, USA) were used for measurements.

### 2.3. Soluble Solids

For soluble solid analysis samples were centrifuged for 10 min at 7200× *g* (Galaxy MiniStar, VWR, Radnor, PA, USA). Soluble solids were determined with a digital refractometer (Pocket Refractometer PAL-1, Atago, Tokyo, Japan) from the resulting supernatant. The results are given as degrees °Brix at 10 ± 0.2 °C.

### 2.4. Particle Size Measurement

The particle size distribution of the samples was determined by static light scattering using a Malvern Mastersizer 3000 (Malvern Instruments, Worcestershire, UK) with an absorption parameter value of 1.5 and refractive index ratio of 1.33. Each sample was diluted with Milli-Q water at 1:50 and mixed for 30–45 min with a magnet mixer. The average d[4.3] and Sauter mean (d[3.2]) corresponding to fine microgel particles are both reported to compare differences in the average volume-weighted and surface weighted particle sizes, respectively. The 90th percentile d[0.9] is also reported to represent the distribution of coarser particles and is used to interpret the sensory perception data as shown in [16,17,24].

### 2.5. Rheological Measurements

The rheological behavior of plant-based and dairy yogurts was characterized by using flow curve, steady shear, and dynamic shear measurements adopted from previous literature [11,16,17,25,26]. All measurements were conducted with a HAAKE MARS 40 Rheometer and monitored by a RheoWin software package, version 2.93 (Thermo Fisher Scientific, Waltham, MA, USA). Samples were analyzed at 10 °C. A cone-plate configuration (cone diameter 35 mm, angle 2°, and gap 0.100 mm) was used in steady shear measurements and flow curves. A plate-plate configuration (diameter 35 mm, gap 1.500 mm) was used in dynamic shear measurements.

#### 2.5.1. Steady Shear Data

The sample (0.4 mL) was placed between cone and plate and then covered with a solvent trap to avoid water evaporation during the resting and measurement. Samples were allowed to rest for 5 min before measurement and a fresh sample was loaded for each measurement. The steady flow properties of each sample were measured at two steady shear rates 10 s^−1^ and 50 s^−1^ [11,16,17,25,26,27]. Viscosity was measured for 120 s while one data point per one second was collected (120 points). In order to understand the thixotropic behavior of the samples, viscosity was plotted against time (s) at constant share rates (10 s^−1^ and 50 s^−1^).

#### 2.5.2. Flow Curves

Flow curves (FCs) were obtained from stepped shear stress ramp between 0.01 s^−1^ and 1000 s^−1^ [25]. The shear rate increased logarithmically for 200 s and then decreased logarithmically for 200 s from 1000 s^−1^ to 0.01 s^−1^. The apparent viscosity was plotted against shear rate to examine the shear thinning behavior. To analyze the recovery of the structure, the area of the hysteresis loop (HL) was determined. Based on the flow curves (between 0.01 and 1000 s^−1^), the consistency index, K, and shear thinning index, n, were calculated using the power law equation (Table 2). Apparent viscosities (ηapp) at shear rates of 10 (s^−1^) from the upward flow curve (Pa·s) were calculated from the Ostwald-de Waele equation.

#### 2.5.3. Dynamic Shear Data

The viscoelastic properties of the samples were studied by strain sweeps and frequency sweeps [11,16,17,25,26]. A plate-plate configuration (diameter 35 mm, gap 1.500 mm) was used in the measurements. The sample (1.5 mL) was placed between the plates and covered with a solvent trap to avoid water evaporation during the resting and measurement. To determine the linear viscoelastic region (LVER), strain sweeps were run at 1 Hz. For the strain sweeps, the step-wise γ increased logarithmically from 0.0001 to 1. The end point of the linear viscoelastic region, thus the point where G′ was 10% lower than the plateau phase of linear viscoelastic region, was measured as stress (G′) and strain (γ). All the frequency sweeps were then performed within the linear viscoelastic region at the following a constant deformation: γ = 0.001 and over the range of f = 0.01–10 Hz. The values of the storage modulus (G′) and the loss modulus (G″) were plotted.

### 2.6. Sensory Analysis

The dynamic mouthfeel perception of the samples was collected by temporal dominance of sensation (TDS) with a consumer test. The participants (*n* = 87) in the study reported consuming either yogurt or yogurt alternatives. A full description of the applied sensory methods, the statistical analysis, and the results can be found in detail in our previous study [6]. According to our previous results, the drivers of mouthfeel liking in plant-based yogurts are thickness and creaminess and the drivers of disliking are wateriness and thinness. These four characteristics were chosen for the present analysis to investigate the physicochemical-mouthfeel relationship. A product average of the dominance durations for each attribute was calculated from the temporal data. The dominance durations are not an approximate visual summary of the panel but represent the average durations of dominant attributes, i.e., for how long each attribute was selected during the mastication. The dominance duration is a recommended parameter to be used when testing product differences in multivariate analysis [28]. It represents the magnitude of the selected attribute among the consumers. Dominance durations have been extracted using left-right standardized individual TDS sequences. This was performed so that panelists with longer perception times would not have more weight in the product means.

### 2.7. Data Analysis

To compare the physicochemical properties between plant-based and dairy yogurts, different parameters were calculated. Steady shear data, flow curves, dynamic strain sweeps, and dynamic frequency sweeps were extracted from the rheological data. In addition, particle size diameters d[3.2], d[4.3], and d[0.9], soluble solids, acidity, and compositional parameters were taken into further analysis. The instrumental data for all parameters measured were examined and determined normally distributed using the Shapiro-Wilk test. One-way analysis of variance was performed on all the instrumental measurements. When the effect was significant, Tukey’s test was applied to determine differences between samples. All analyses were performed in triplicate using SPSS version 25 (SPSS Inc., Chicago, IL, USA).

In order to visualize which of the physicochemical and previously studied mouthfeel sensations contribute most to the differences between plant-based and dairy yogurts, principal component analysis (PCA) was conducted. PCA is a procedure that examines the relationships among a set of correlated variables. The obtained results were visualized graphically by projecting the samples (scores) and physicochemical as well as mouthfeel variables (loadings) onto the space defined by the two first PCs.

To determine if the previously studied mouthfeel sensations (thick, thin, creamy, and watery) could be explained by physicochemical properties in plant-based yogurts, a relationship between two datasets among plant-based yogurts was summarized and visualized by partial least squares regression (PLS-R). In addition, Pearson correlations were analyzed to support the results of the PLS-R. All extracted physicochemical parameters and mouthfeel sensations (thick, creamy, thin, and watery) were included for the analysis. PLS regression is designed to determine relationships existing between dependent (Y, mouthfeel sensations) and explanatory (X, physicochemical properties) variables by seeking underlying factors common to both sets of variables [29]. The model was developed using internal cross-validation based on y, mouthfeel sensations, and X, physicochemical properties. PLS-R is a suitable model because it allows for small to medium sample sizes, a large number of independent variables, and is robust to multicollinearity. Both PCA and PLS were analyzed using Unscrambler (Unscrambler 7.6 SR-1, Camo Asa, Oslo, Norway).

## 3. Results

### 3.1. Acidity

The acidity differences between the instrumental and sensory batches were small, indicating similarities between the batches (Table 3) and thus validating their comparison. The pH in both dairy and nondairy samples ranged from 3.4 and 4.4, with one sample (P4) having a significantly lower pH (3.4) compared to the other samples. The total titratable acidity showed clear differences between dairy and plant-based samples. Dairy yogurts had significantly higher TTA compared to plant-based yogurts, and P2 and P3 had the lowest TTA, 2.20 and 2.18, respectively.

### 3.2. Soluble Solids

**Figure 1:**
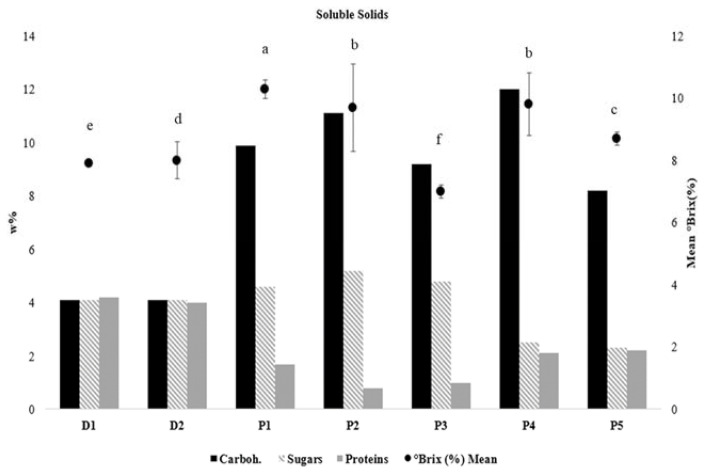
The final carbohydrate, sugar, and protein content as labelled in the products (w-%) and °Brix (%) with standard deviation. Superscript letters indicate statistical difference in °Brix (%) between the samples (*p* < 0.05).

Figure 1 shows the calculated °Brix with the carbohydrates, sugars, and proteins that are obtained from the label information. The soluble solids (°Brix, %) ranged from 7.0 to 10.3 between all the samples, with P1 and P4 having the highest while P3 and P5 having the lowest °Brix among the plant-based samples. The figure demonstrates that samples with higher total carbohydrate content (P1, P2, and P4) also have the highest °Brix values.

### 3.3. Particle Size Measurements

The smallest particles by diameter were discovered in sample P4 (d[3.2] = 14 µm) (Table 4). The d[3.2] values ranged from 14 to 36 µm and 20 to 21 µm in plant-based and dairy yogurts, respectively. The d[4.3] values ranged from 22 to 68 µm and 27 to 28 µm in plant-based and dairy yogurts, respectively. The d[0.90] values ranged from 42 to 151 μm and 47 to 52 μm in plant-based and dairy yogurts, respectively. Sample P5 had the greatest particle size (d[0.90] = 151 μm) among all the samples. Compared to other plant-based samples, P3 had the most similarities with dairy yogurts in particle size and diameters.

**Table 4:** Particle size diameters (±standard deviation) of all the samples. Superscript letters indicate statistical difference in the same row (*p* < 0.05).

	D1	D2	P1	P2	P3	P4	P5
d[3.2] + s.d. (µm)	20 ± 0.2 ^bc^	21 ± 0.3 ^b^	15 ± 0.1 ^e^	36 ± 0.4 ^a^	20 ± 0.1 ^c^	14 ± 0.1 ^f^	19 ± 0.1 ^d^
d[4.3] + s.d. (µm)	27 ± 0.2 ^d^	28 ± 0.9 ^c^	24 ± 0.5 ^e^	48 ± 0.1 ^b^	30 ± 0.4 ^c^	22 ± 0.3 ^f^	68 ± 1.2 ^a^
d[0.9] + s.d. (µm)	47 ± 0.6 ^e^	52 ± 2.2 ^cd^	48 ± 1.4 ^de^	76 ± 0.7 ^b^	56 ± 0.7 ^c^	42 ± 0.4 ^f^	151 ± 3.5 ^a^

### 3.4. Rheological Measurements

#### 3.4.1. Steady Shear Data

**Figure 2:**
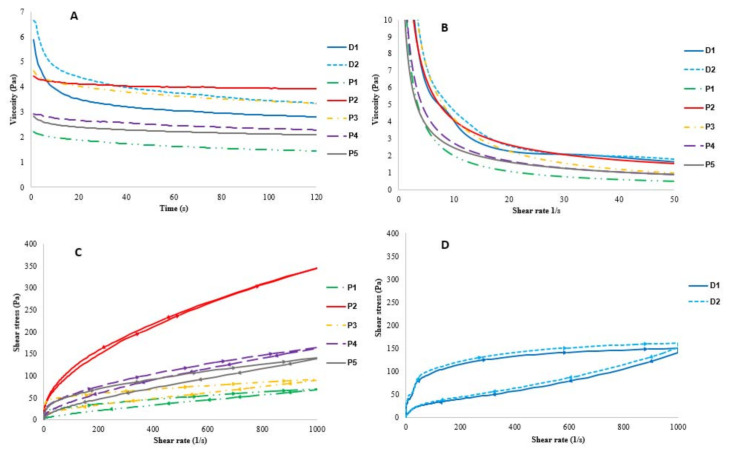
(**A**) Viscosity (Pa·s) during 120 s at a steady shear rate of 10 s^−1^. (**B**) Flow curve: viscosity (Pa·s) by shear rate (s^−1^). (**C**) Hysteresis loops in plant-based yogurts. (**D**) Hysteresis loops in dairy yogurts.

Different parameters help to articulate discernable rheological differences among samples (Table 5). All the samples showed thixotropic behavior at steady shear rates (10 s^−1^ and 50 s^−1^), thus demonstrating structural breakdown under flow. For most of the samples, the viscosity decreased rapidly at the beginning of the measurement and then decreased slowly, staying nearly constant (Table 5). The dairy yogurts had a stronger decline in their viscosity than in the plant-based samples. Particularly at shear rates of 5 and 10 s^−1^, samples P2 and P3 showed similar behavior to dairy yogurts compared to other plant-based samples (Figure 2B). The viscosity of sample P2 remained nearly constant after the first drop at the beginning of the measurement (Figure 2A). Yet, a higher shear rate was associated with a lower viscosity also for P2.

**Table 5:** The mean value of the rheological parameters of all the samples. Superscript letters indicate statistical differences in the same row (*p* < 0.05).

	D1		D2		P1		P2		P3		P4		P5	
SS10 (Pa s)	3.92	±0.20 ^b^	4.82	±0.17 ^a^	1.99	±0.05 ^d^	4.20	±0.06 ^b^	4.20	±0.03 ^b^	2.76	±0.06 ^c^	2.50	±0.06 ^c^
SS50 (Pa s)	1.53	±0.05 ^b^	1.99	±0.11 ^a^	0.52	±0.01 ^e^	1.55	±0.00 ^b^	1.01	±0.01 ^c^	0.90	±0.01 ^c d^	0.85	±0.01 ^d^
HL (-)	57,416.48	±1479.05 ^b^	59,720.44	±1242.04 ^a^	10,937.42	±148.72 ^d^	−4647.42	±152.23 ^e^	17,678.42	±291.84 ^c^	11,022.59	±177.27 ^d^	16,278.60	±22.35 ^c^
n (-)	0.31	±0.01 ^b^	0.28	±0.01 ^c^	0.15	±0.01 ^d^	0.35	±0.00 ^a^	0.15	±0.02 ^d^	0.31	±0.00 ^b c^	0.36	±0.01 ^a^
K (Pa s ^n^)	21.15	±1.65 ^b^	26.00	±2.06 ^a^	14.02	±0.28 ^c^	18.91	±0.15 ^b^	27.94	±1.18 ^a^	13.52	±0.08 ^c d^	10.73	±0.09 ^d^
ηapp10 (1/s)	4.35	±0.27 ^b^	4.94	±0.29 ^a^	1.99	±0.02 ^d^	4.27	±0.03 ^b^	3.97	±0.02 ^b^	2.75	±0.01 ^c^	2.45	±0.03 ^c^
G’LVE (Pa)	302.00	±15.46 ^b^	380.71	±37.11 ^a^	59.08	±3.41 ^d e^	77.73	±1.39 ^d^	195.05	±12.77 ^c^	16.48	±0.92 ^e^	24.90	±2.35 ^e^
γLVE (-)	0.01	±0.00 ^c^	0.01	±0.00 ^c^	0.02	±0.00 ^c^	0.03	±0.00 ^b c^	0.07	±0.02 ^a^	0.06	±0.00 ^a b^	0.06	±0.01 ^a^
G′ (Pa)	303.30	±14.57 ^a^	431.60	±13.47 ^b^	61.22	±5.45 ^e^	89.30	±1.01 ^d^	226.15	±1.75 ^c^	17.69	±1.58 ^f^	25.67	±1.10 ^f^
G″ (Pa)	74.60	±2.52 ^a^	102.88	±0.81 ^b^	7.21	±0.27 ^d e^	23.13	±0.12 ^d^	14.81	±0.06 ^c^	8.53	±0.67 ^e^	10.51	±0.23 ^e^

#### 3.4.2. Flow Curves

All the samples showed shear thinning behavior (*n* < 1) as the apparent viscosity decreased by increasing the shear rate (Figure 2B) in all the samples (Table 5). The thixotropic properties were measured by calculating the hysteresis loops, i.e., the area between the forward and backward curves (Figure 2C,D). A greater area within the hysteresis loops was reported with dairy yogurts (D1-D2) compared to other yogurts. Furthermore, for sample P2, the hysteresis loop showed the following different behavior compared to other samples: the forward and backward curves were partly overlapping (within 500–1000 s^−1^), the backward curve being also partly higher than the forward curve, indicating reversible shear-thinning behavior (Figure 2C).

#### 3.4.3. Dynamic Shear Data

**Figure 3:**
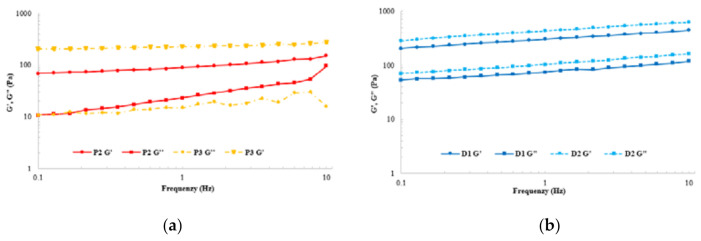
An example of the viscoelastic properties of both types of the following samples: plant-based samples P2 and P3 in Figure (**a**) and dairy samples in Figure (**b**).

Frequency sweeps showed that elastic properties dominated in the linear viscoelastic area. Examples of the viscoelastic properties of samples P2 and P3 as well as the dairy samples are shown in Figure 3. There are significant disparities among the samples in the storage modulus, indicating that the samples represent a wide range of texture properties, particularly in rigidity. All samples had G′ > G″ and thus can be described as soft fluid gels (Table 5). The storage modulus of the dairy samples as well as samples P2 and P3 was significantly higher than the storage modulus for other samples, indicating a more rigid structure compared to other products. This could be due to a high fat content in samples P2 and P3, 2.4 and 2.4 g/100 mL, respectively. Sample P3, however, had the lowest storage modulus, while also the lowest fat content, at 0.8 g/100 mL.

### 3.5. Physicochemical Differences in Dairy- and Plant-Based Yogurts

**Figure 4:**
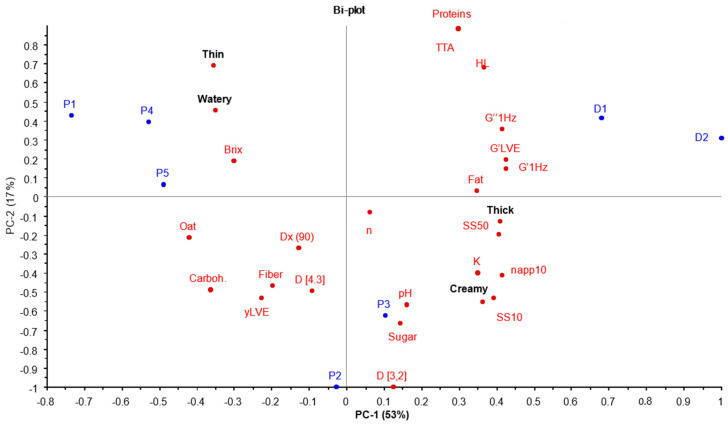
PCA biplot (scores and loadings) of the physicochemical properties for plant-based and dairy yogurts plus the following mouthfeel sensations: thick, creamy, thin, and watery. The abbreviations of the physicochemical parameters are in accordance with Table 2.

A PCA analysis was applied to demonstrate the positioning of the plant-based and dairy yogurts when the average values of the instrumental and mouthfeel properties were applied. The first two components accounted for 70% of the total variability. The biplot graph (Figure 4) visualizes the similarities and differences between the products in physicochemical and mouthfeel properties. The first component, which explained the higher percentage of variability (53%), separated the samples clearly according to their viscosity, including both large and small deformation tests. Products P1, P4, and P5 were in the negative part of the first component, dairy products were in the positive part of the component, and products P2 and P3 were in the middle. The second component, which accounted for 17% of the variability, separated P2 and P3 from the other samples, at least according to the differences in steady shear viscosity compared to the other samples. In addition, samples were separated by negative and positive mouthfeel sensations, which were placed on opposite sides of the scale in both of the PCs. Furthermore, the rheological parameters and the PCA graph indicate a pattern between the following large and small deformation tests: Large deformation tests correlate positively with plant-based yogurts P2 and P3, whereas small deformation tests represent dairy yogurts. 

### 3.6. Physicochemical and Mouthfeel Properties among Plant-Based Yogurts

**Figure 5:**
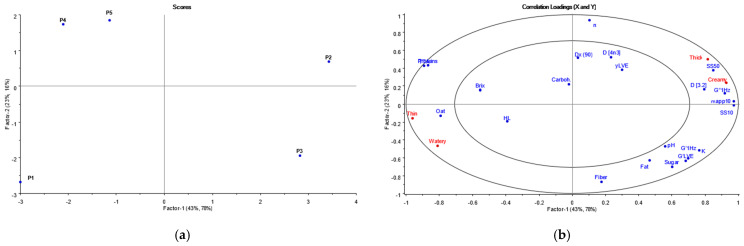
PLS regression bi-plots for scores (**a**) and for loadings (**b**) of sensory and physicochemical parameters for five plant-based yogurts. The abbreviations of physicochemical parameters are in accordance with Table 2.

The relationship between the mouthfeel attributes and the physicochemical properties among plant-based yogurts was studied and visualized by PLS regression (Figure 5). Mouthfeel sensations used in the PLS regression figure represent the dominance durations for each attribute and thus describe the magnitude of each attribute. The first factor of the PLS regression model explained 43% of the variation in the physicochemical results and 78% of the variation in the sensory data within the five samples analyzed. The second factor explained 23% and 16%, respectively. Altogether, the PLS regression model explained 66% of the variation in the instrumental and 94% of the variation in the mouthfeel using the two first principal components, i.e., the variation in the samples is explained more specifically by sensory results than by instrumental results, according to the two components. However, the relatively large explanation rate in both results suggests that instrumental and sensory analyses are complementary methods. To determine which of the parameters had a significant correlation with the mouthfeel sensations, Pearson correlations were studied among the plant-based samples (Table A1).

## 4. Discussion 

### 4.1. Rheological Measurements

The flow behavior index (n) values were consistent with those in previous literature, with almond yogurt showing the most similarities with oat yogurts [21], while the consistency coefficient (K) values of the studied plant-based yogurts were different to those of soy, coconut, cashew, almond, and hemp yogurts [21,22]. The greater area within the hysteresis loops reported with dairy yogurts (D1-D2) suggests stronger thixotropic behavior, which can be interpreted as a more permanent structure breakdown. This is a well-known behavior in dairy yogurts [30]. Plant-based products, however, showed visible yet significantly smaller hysteresis loop areas compared to dairy yogurts, indicating a faster structure recovery over time. This is most likely due to the difference in the gelling agents and the differences in their interactions. In plant-based yogurts, the closely packed polysaccharides can partly reform the structural network by means of noncovalent interactions. Another study showed similar results after the shearing of fermented oat-based gels, which were able to partially recover their initial structure [2]. They suggested that the higher total solid content contributes to more junction zones present between the particles, which results in the faster rearrangement of the microstructure. Our results support the same with soluble solids samples with the highest °brix (P1 and P4) had the smallest hysteresis loops, and samples with the lowest °brix had the largest hysteresis loops.

Furthermore, it has been demonstrated with oat-starch gels that the rate of the disentanglement of the macromolecules was higher than their re-entanglement during the shearing [31]. This results in a visible hysteresis loop and applies to the dairy and the majority of the plant-based yogurts in our study. In addition to the overlapping curves in hysteresis loops for sample P2, it also showed a significantly higher shear stress than the other products, reflecting its higher resistance to shear forces. The counterclockwise loop could be explained by the higher amount of remaining beta-glucan in the sample, thus contributing to the thickening behavior more effectively compared to other oat-based products. However, this interaction is not possible to discuss further, as the beta-glucan content of the samples was not analyzed. Sample P2 also contained modified starch, which could be a contributor to the different hysteresis loops. A similar pattern has been shown with solutions containing amylodextrin and beta-glucan [32].

Sample P3, however, had the lowest storage modulus, also the lowest fat content, at 0.8 g/100 mL. This is supported by another study which suggests that for a mixed food system such as fermented oat-based gel, it is likely that swollen starch granules, protein aggregates, and residual small fat droplets act as the fillers and are able to increase the rigidity (G′) of the system [2]. In addition, it has been demonstrated with polysaccharide gels that the gel-like behavior is related to molecular and physical interactions and thus the formation of the network structure [33]. It is, therefore, likely that added hydrocolloids contributed to the viscoelastic properties. Taking this into account, samples P2 and P3 were the only plant-based samples containing pectin in addition to starch. The loss moduli were also the highest for the thickest plant-based samples, P2 and P3, indicating stronger viscous behavior. At the endpoint of a linear viscoelastic area, stress (G’LVE) discriminated the samples more than the strain (γLVE) did.

### 4.2. The Physicochemical Dividers between Dairy and Plant-Based Yogurts

The most salient difference between plant-based and dairy yogurts is in the macromolecules that form their structures. The PCA biplot demonstrates how the macromolecules divide the samples according to their protein, fat, and carbohydrate contents. The lower protein content in plant-based yogurts (0.8 to 2.2 w-%) compared to dairy yogurts (4.0–4.2 w-%) may be seen in the lower viscosities at the beginning of the steady shear measurement, indicating a weaker initial structure. Even if bovine β-lactoglobulin has been shown to have a critical concentration for the sol-gel transition at 1%, as suggested by [34], the protein concentration in spoonable yogurts is typically greater than 3%. In the dairy yogurts of this study, the protein content was enough to build structures comparable to those obtained by various thickeners in the plant-based yogurts. All the plant-based products contained added thickeners, namely, potato, corn, tapioca starch, pectin, xanthan, or locust bean gum, which are the main contributors to the viscosities in plant-based gels. Furthermore, starch and cell wall polysaccharides are present in different amounts depending on the oat ingredient used [2,3,4].

### 4.3. Relationship between Physicochemical and Mouthfeel Properties among Plant-Based Yogurts

The PLS regression demonstrates that thickness and creaminess are associated with each other, consistent with prior studies indicating that creaminess results from a thick mouthfeel [35,36]. Moreover, increased viscosity has been linked with creaminess in dairy yogurts [10,35]. Our results indicate that of all the physicochemical parameters, rheological parameters showed the strongest connections with thickness and creaminess, particularly in large deformation tests in plant-based yogurts. Pearson correlations also support this; all the mouthfeel sensations are correlated with both steady shear rates (SS10 and SS50) and apparent viscosity either positively (thick and creamy) or negatively (thin and watery). Previously, the a shear rate of 50 s^−1^ has been regularly adopted by many authors with semisolids [27,36,37]. Positive correlations have been found with both 50 s^−1^ and 100 s^−1^ [16], as well as with mouthfeel thickness and 100 s^−1^ in dairy yogurts [14]. According to Pearson correlations, both shear rates are connected with all four mouthfeel sensations.

According to the PLS regression visualization, among small deformation tests, only the loss modulus (G″) was connected with thickness and creaminess. This indicates that the viscous properties are more strongly connected to the thickness and creaminess than the elastic properties. By contrast, the elastic properties (G′) have been associated with a viscous and fatty mouthfeel in dairy yogurts [16]. Our results indicate that fat content is associated with G′ in plant-based yogurts (Figure 5); however, the fat content or G′ do not describe the thickness or creaminess in the studied plant-based yogurts as much as they describe the thickness and creaminess in dairy yogurts (Figure 4 and Figure 5). It has also been demonstrated that fat content is connected with a creamy and thick mouthfeel in dairy yogurts [38,39,40]. These differences between dairy- and plant-based yogurts could be due to the differences in fat content between the yogurts and the milk fat crystals melting in the mouth, which may contribute to the creamy mouthfeel, whereas, in plant-based yogurts, the canola and rapeseed oils are in liquid form. It has also been suggested that a creamy mouthfeel in dairy yogurts is strongly associated with the coalescence of emulsion droplets in the mouth and with the spreading of released fat at oral surfaces [41,42]. There is, however, previous evidence on the creamy mouthfeel in the following plant-based gels: an oat gel with a higher total solids content was perceived as creamier compared to a gel with a lower total solids content [2]. Furthermore, it has been suggested that structural components such as starch and protein aggregates create a smooth and thick mouthfeel in the absence of milk fat [36]. Another study with dairy and plant-based yogurts demonstrated that a high protein content provided a better gel firmness and a higher consistency coefficient (K) [21]. In addition, added starch in dairy yogurts has been shown to increase consistency, creaminess, and overall liking [43]. According to the PLS, the particle size parameter d[3.2] was associated with thick and creamy mouthfeel, whereas the other particle size parameters were not associated with any of the mouthfeel sensations. Previous literature suggests that a small particle size explains creaminess in dairy yogurts [14,16,24,26,36]. However, our results indicate that with a particle size d[3.2] of ≥20 µm, there is a connection to thickness and creaminess.

Both the PLS regression and the PCA graphs demonstrate that thin and watery are similar properties in plant-based yogurts (Figure 4 and Figure 5). One explanation could be that the panelists were not able to distinguish wateriness from thinness. Another explanation could be that a watery mouthfeel is a consequence of the hydrolysis of starch by α-amylase, which present in saliva [44]. Our previous results support this: watery was perceived mainly after thinness [6]. The PLS graph and Pearson correlation indicate that thin and watery correlated negatively with fat content. A similar correlation has been demonstrated within emulsion-filled gels [20]. They showed that low-fat content relates to wateriness and that watery is the opposite of creamy. Additionally, as more saliva is added to the bolus, the perceived attributes have been found to relate to consistency (e.g., creaminess and wateriness) [8]. Furthermore, similar results were found with a descriptive analysis [45]. They concluded that the watery mouthfeel in semisolid gels is a chew-down property, whereas, sugar reduction in yogurts has been shown to result in a watery mouthfeel [19]. This should be investigated further. Interestingly, our results indicate that the samples with the lowest sugar content (P4 and P5) were perceived as watery, whereas samples with the highest sugar content (P2 and P3) were perceived as creamy.

## 5. Conclusions

There is a growing requirement for plant-based yogurts that meet consumer demands in terms of texture. Extensive previous literature demonstrates the relationship between physicochemical and mouthfeel properties in conventional dairy yogurts. However, more research is required on plant-based yogurts. The aim of the study was to determine the physicochemical properties of different commercial plant-based yogurts. The results were compared to those for dairy yogurts and previously studied mouthfeel sensations.

Plant-based yogurts exhibited a wide range of viscoelastic properties, which was a result of the fact that different hydrocolloids at different levels were incorporated in the samples at different levels. Our results also revealed some structural differences in the following two product groups: for example, a significantly stronger structure recovery was found in plant-based yogurts than in dairy yogurts, resulting from the differences in the gelling agents and their interactions. This study demonstrates that dairy and plant-based yogurts with a similar mouthfeel profile may have different viscoelastic properties. The considerable physicochemical differences between the two product groups are likely to also be valid with other similar yogurts as the selected samples in this study represent the typical dairy- and plant-based yogurts in the market. Further investigation is necessary to demonstrate this.

This study highlights the importance of rheological large deformation tests and their ability to explain essential mouthfeel sensations in plant-based yogurts. Thick and creamy mouthfeel sensations were positively correlated with steady shear rates and apparent viscosity. The results also suggest that oil content does not significantly affect creaminess in plant-based yogurts. The results emphasize that instrumental and sensory methods should not be considered substitutive but complementary methods when developing plant-based yogurts in a cost-effective and timely manner.

### Limitations and Future Challenges

The presented relationships between the physicochemical parameters and mouthfeel are only valid within the studied plant-based yogurts. The results highlight that further investigation is necessary to demonstrate the impact of different macromolecules and hydrocolloids on the physicochemical and sensory properties in plant-based yogurts. 

## Figures and Tables

**Table 1:** The bases, thickeners, stabilizers, and oils as declared on the labels of all the samples.

	Base	Thickener	Stabilizer or Preservative	Oil (g/100 mL)
D1	Dairy	None	None	Milk fat (2.5)
D2	Dairy	None	None	Milk fat (4)
P1	Oat base (water, oat 12%), potato protein	Potato starch	Calcium carbonate (E170), Tricalcium phosphate (E341)	Rapeseed oil (2.2)
P2	Oat base (water, oat 8.5%)	Modified starch, pectin	Potassium sorbate (E202)	Canola oil (2.4)
P3	Oat base (water, oat flakes 8%)	Starch (corn, potato), pectin	Tricalcium phosphate (E341)	Canola oil (2.5)
P4	Water, oat 12%, and potato protein	Starch (tapioca, potato), xanthan, and locust bean gum	None	Canola oil (0.8)
P5	Oat base (water, oat 8.2%), pea protein	Modified potato starch	None	Canola oil (0.9)

**Table 2:** Overview of the physicochemical parameters extracted from instrumental measurements.

Type of Measurement	Explanation	Codes
Large deformation test:	η at 10 s^−^¹ at t = 10 s.	SS10
Steady shear rate (SS)	η at 50 s^−^¹ at 10 s.	SS50
	The area of the hysteresis loop between the upward and downward curves	HL
Large deformation test: Flow curves (FCs)	Shear thinning index, n, and consistency, K, were calculated from the power law (η = K*ẏn¹) from the upward flow curve	n, K
	Apparent viscosities (η_app_) from upward flow curve (Pa·s) calculated from Ostwald-de Waele = Kẏ^(n^−^¹) at shear rates 1.5, 5, 10, 25, and 50 (1/s)	ηapp10
Small deformation test:	Stress (G′) at the end point of LVER	G’LVE
Dynamic strain sweeps (DSSs)	Strain (γ) at the end point of LVER	γLVE
Small deformation test:	G′ at 1 Hz, Pa (DFS G′1 Hz)	G′
Dynamic frequency sweep (DFS)	G″ at 1 Hz, Pa (DFS G″1 Hz)	G″
Particle size	Surface weighted particle size	d[3.2]
	Volume weighted particle size	d[4.3]
	90th percentile of the particles less than d[0.9]	d[0.9]
Chemical composition	Fat content	Fat
	Carbohydrate content	Carboh.
	Sugar content	Sugar
	Fiber content	Fiber
	Protein content	Proteins
	Oat content	Oat
Soluble solids	°Brix	°Brix
Acidity	pH	pH
	Total titratable acidity	TTA

**Table 3:** pH and TTA of all the samples in the instrumental analysis (±standard deviation) and difference to the samples in the sensory analysis. Superscript letters indicate statistical difference between the samples, in the same column (*p* < 0.05).

	pH		TTA	
	Instrumental Analysis ± STD	±Sensory Analysis	Instrumental Analysis ± STD	±Sensory Analysis
D1	4.27 ± 0.12 ^bc^	−0.06	10.83 ± 0.09 ^a^	−0.18
D2	4.18 ± 0.12 ^bc^	−0.12	10.86 ± 0.10 ^a^	0.22
P1	4.16 ± 0.08 ^c^	0.01	4.43 ± 0.14 ^c^	−0.09
P2	4.26 ± 0.10 ^b^	−0.07	2.00 ± 0.08 ^d^	−0.05
P3	4.43 ± 0.11 ^a^	−0.17	2.18 ± 0.16 ^d^	0.14
P4	3.47 ± 0.12 ^d^	0.06	5.36 ± 0.45 ^b^	0.23
P5	4.26 ± 0.11 ^bc^	−0.08	5.54 ± 0.33 ^b^	0.21

## Data Availability

Data is contained within the article.

## Appendix A

**Table A1:** Pearson correlations between physicochemical and mouthfeel parameters in plant-based yogurts (*n* = 5). Correlation coefficients in bold are significant at *p* < 0.05 (*) and at *p* < 0.01 (**).

	Creamy	Thick	Thin	Watery
HL	−0.188	−0.541	0.260	0.373
n	0.295	0.572	−0.219	−0.466
K	0.634	0.391	−0.716	−0.493
ηapp10	**0.893 ***	0.878	**−0.955 ***	**−0.884 ***
SS10	**0.908 ***	0.846	**−0.966 ****	−0.873
SS50	0.813	**0.964 ****	−0.846	**−0.894 ***
G’LVE	0.562	0.215	−0.619	−0.318
γLVE	0.582	0.396	−0.527	−0.557
G′1 Hz	0.584	0.242	−0.640	−0.346
G″1 Hz	0.812	0.857	−0.847	−0.771
D [3.2]	0.668	0.767	−0.692	−0.633
D [4.3]	0.430	0.297	−0.281	−0.199
Dx (90)	0.283	0.108	−0.116	−0.043
°Brix	−0.684	−0.272	0.646	0.418
Fat	0.153	0.205	−0.314	−0.228
Carboh.	−0.153	0.322	0.021	−0.299
Sugar	0.272	0.181	−0.376	−0.128
Fiber	−0.257	−0.217	0.142	0.328
Protein	−0.625	−0.546	0.717	0.516
Oat	**−0.893 ***	−0.594	0.818	0.592
pH	0.477	0.064	−0.428	−0.028
TTA	−0.670	−0.559	0.757	0.544
